# P-782. Nontuberculous Mycobacterial Infections among Lung Transplant Recipients: A Nine-Year Cohort Study

**DOI:** 10.1093/ofid/ofae631.976

**Published:** 2025-01-29

**Authors:** Keiko Soneda, Shinya Yamamoto, Koh Okamoto, Yoshimi Higurashi, Kazuhiko Ikeuchi, Mahoko Ikeda, Shu Okugawa, Mitsuaki Kawashima, Chihiro Konoeda, Masaaki Sato, Takeya Tsutsumi

**Affiliations:** Tokyo University Hospital, Bunkyo-ku, Tokyo, Japan; Tokyo University Hospital, Bunkyo-ku, Tokyo, Japan; The University of Tokyo Hospital, Bunkyo, Tokyo, Japan; The University of Tokyo Hospital, Bunkyo, Tokyo, Japan; Tokyo University Hospital, Bunkyo-ku, Tokyo, Japan; Tokyo University Hospital, Bunkyo-ku, Tokyo, Japan; The University of Tokyo Hospital, Bunkyo, Tokyo, Japan; Tokyo University Hospital, Bunkyo-ku, Tokyo, Japan; Tokyo University Hospital, Bunkyo-ku, Tokyo, Japan; Tokyo University Hospital, Bunkyo-ku, Tokyo, Japan; The University of Tokyo Hospital, Bunkyo, Tokyo, Japan

## Abstract

**Background:**

The epidemiology, microbiology, and clinical course of nontuberculous mycobacterial (NTM) infection among lung transplant recipients vary considerably by geographic location. Their data is still scarce.

**Figure:**
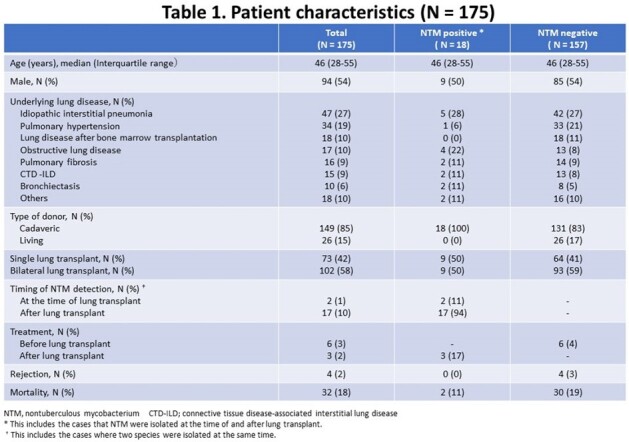

**Methods:**

This single-center retrospective study was conducted at one of the major transplant centers in Japan. Medical records and microbiological test results of all the lung transplant recipients who underwent lung transplants between 2015 to 2023 were reviewed through December 31, 2023. Per institutional protocol, tracheal aspirates or sputum have been sent for mycobacterial cultures at the time of transplant and when clinically indicated thereafter. The definition of pulmonary NTM disease was based on the ATS/ERS/ESCMID/IDSA guidelines.

**Figure:**
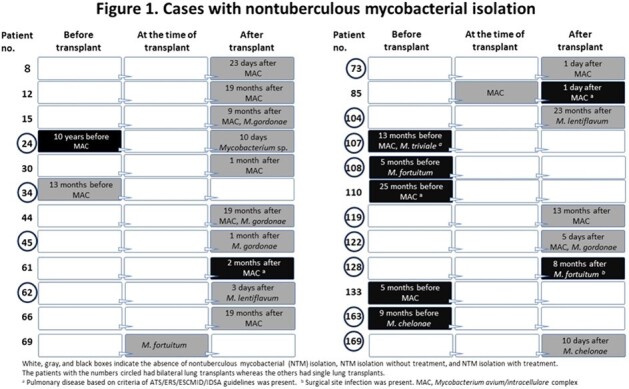

**Results:**

In total, 175 lung transplants were performed during the study period (Table 1). Before transplant, 7 recipients had NTM isolation from the sputum and 5 of these 7 recipients had received bilateral lung transplants. While only 2 of these 7 recipients met the diagnosis of NTM pulmonary disease, 6 recipients received antimycobacterial treatment for a median duration of 25 months before transplant and only one case had NTM isolation after transplant (Figure 1). At the time of lung transplant, 169/175 (97%) recipient’s sputum were collected. Six species of NTM were isolated in 18 patients (10%, 18/175) at various times; at the time of transplant, between 0-1 month, 1-6 months, 6-12 months, and after 12 months in 2, 7, 3, 2, and 5 recipients, respectively (Figure 1, 2). Twenty-two isolates were recovered at the time of and after transplant, slowly growing mycobacteria accounted for 86% (19/22), including *M. avium* complex (N = 12) and *M. gordonae* (N = 4), whereas rapidly growing mycobacteria, *M. fortuitum* (N = 2) and *M. chelonae* (N = 1), accounted for the rest. Although 2 of 18 recipients with NTM isolation died, one recipient died directly from NTM infection complicated by *Aspergillus* coinfection.

**Figure:**
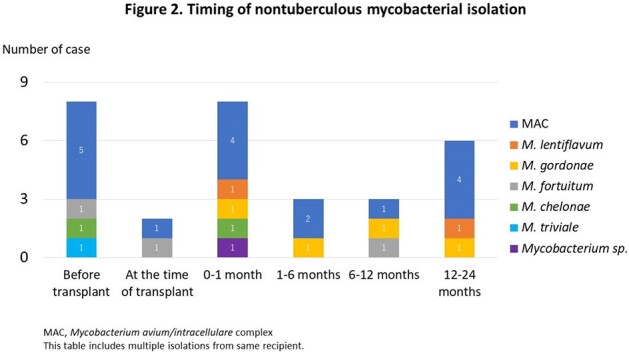

**Conclusion:**

NTM were isolated in 10% of recipients at the time of and after lung transplant. NTM, isolated before and at the time of transplant, rarely required treatment after transplant.

**Disclosures:**

**All Authors**: No reported disclosures

